# 

**Published:** 1983

**Authors:** 


Contents

1. Editorial
4. Some Famous Bristol Doctors
C. Bruce Perry
18. The Fleece Medical Society
H. J. Eastes
23. Familial Mental Handicap
J. Jancar
28. Surgery of the Circulation
R. Baird
33. The Treatment of Pancreatitis
A. M. N. Gardener and N. Setakis
37. From Our Correspondents
40. The Surgical Club of South West England
Abstracts of papers given at the Autumn Meeting in Bath
45. Miscellanea
Dr. T. Daureeawoo
'A crabbit old woman wrote this'
Clerihews
'Don't forget to bring your cheque book'
47. Examination Results

